# Smoking, alcohol and gastric cancer risk in Korean men: the National Health Insurance Corporation Study

**DOI:** 10.1038/sj.bjc.6603893

**Published:** 2007-07-17

**Authors:** N Y Sung, K S Choi, E C Park, K Park, S Y Lee, A K Lee, I J Choi, K W Jung, Y J Won, H R Shin

**Affiliations:** 1 National Cancer Control Research Institute, National Cancer Center, Gyeonggi-Do, Korea; 2 Health Insurance Research Center, National Health Insurance Corporation, Seoul, Korea; 3 Research Institute and Hospital, National Cancer Center, Gyeonggi-do, Korea

**Keywords:** cigarette smoking, alcohol intake, gastric cancer, subsite

## Abstract

We investigated the risk of gastric cancer by subsite in relation to cigarette smoking and alcohol in a large population-based cohort of 669 570 Korean men in an insurance plan followed for an average 6.5 years, yielding 3452 new cases of gastric cancer, of which 127 were cardia and upper-third gastric cancer, 2409 were distal gastric cancer and 1007 were unclassified. A moderate association was found between smoking, cardia and upper-third (adjusted relative risk (aRR) 2.2; 95% confidence interval (CI) 1.4–3.5) and distal cancers (aRR=1.4; 95% CI=1.3–1.6). We also found a positive association between alcohol consumption and distal (aRR=1.3; 95% CI=1.2–1.5) and total (aRR=1.2; 95% CI=1.1–1.4) gastric cancer. Combined exposure to high levels of tobacco and alcohol increased the risk estimates further; cardia and upper-third gastric cancers were more strongly related to smoking status than distal gastric cancer.

Until recently, gastric cancer was the second most common cancer worldwide; now, however, with an estimated 934 000 new cases (8.6% of new cancer cases) in 2002 alone, it is in fourth place behind lung, breast and colon and rectum cancers (Parkin *et al*, 2005). Although declining in Korea, gastric cancer is still the commonest cancer (Shin *et al*, 2005b).

An extensive review indicates that smoking is a moderate risk factor for gastric cancer (International Agency for Research on Cancer, 2004), but little support exists for an association with alcohol (Gammon *et al*, 1997; Sjödahl *et al*, 2006). The possibility of a differential effect of smoking and alcohol consumption on different gastric subsites, however, remains to be clarified.

In a prospective cohort study, we investigated the effects of smoking and alcohol consumption on the risk of gastric cancer by subsite in the National Health Insurance Corporation Study (NHICS).

## MATERIALS AND METHODS

The NHICS is a cohort investigation that was designed to assess the risk factors for the incidence of and mortality from cancer (Yun *et al*, 2005; Park *et al*, 2006). In brief, the cohort consisted of government employees, teachers and their dependents who were insured by the Korea National Health Insurance (NHI) Program in 1996, had at least one medical examination, and completed a self-administered questionnaire.

The study participants were derived from 692 108 men aged 30 years or over who participated in the National Health Examination Program in 1996 and were in the NHICS cohort. Of the 692 108 participants, we excluded 2732 patients who had cancer at enrollment according to the Korea Central Cancer Registry (KCCR). We also excluded the following because of missing information: 214 for weight or height, 9936 for smoking, 3019 for alcohol intake and 6573 for dietary preference. Ultimately, 669 634 participants were included.

Based on questionnaire responses at the baseline examination of the NHICS cohort, the participants were classified as ‘current smokers’ if they reported smoking currently for at least 1 year, ‘nonsmokers’ if they never smoked and ‘former smokers’ if they had smoked but quit. Current smokers were further classified by the average number of cigarettes smoked per day (1–19, ⩾20) and duration of smoking (1–19, 20–29, or ⩾30 years). Alcohol intake per day was categorised as follows: no drinking (0 g), light drinking (1–14.9 g), moderate drinking (15.0–24.9 g) and heavy drinking (⩾25.0 g). Total daily alcohol intake was expressed as the number of glasses per week of Korea's most popular alcoholic beverage, ‘Soju’. One glass of Soju contains about 12 g of ethanol. A preference for saltiness in food (low salt, normal and salty) was included because of possible relevance to stomach cancer (Tsugane *et al*, 2004). We used the World Health Organisation body mass index (BMI) standards for Asians (World Health Organisation, 2000).

The principal outcome variable was incident gastric cancer cases identified from the KCCR, a nationwide hospital-based system that includes 94% of the country’s university hospitals and 96% of the resident training hospitals; it covers at least 90% of the newly diagnosed malignancies in Korea (Ministry of Health and Welfare, 2002). Using the KCCR, we identified 3516 men who were diagnosed with gastric cancer from 1996 to 2002, from which we excluded the 64 people with multiple primary cancers.

We used anatomic site and histological classification information from the pathology reports of the KCCR based on the International Classification of Diseases for Oncology. Tumours at the oesophagogastric junction or upper third of the stomach were classified as cardia and upper-third gastric cancer (C16.0–16.1) and those at the lower end of the stomach as distal gastric cancer (C16.2–16.7). Mixed site (C16.8) and site not otherwise specified (C16.9) were regarded as unclassified. Virtually all (>98%) of the gastric cancers were confirmed histologically, 94% being adenocarcinoma (M814–857), and the few cases (*n*=212) that were not excluded. During the 6.5-year follow-up period, we included 3452 patients diagnosed with a gastric adenocarcinoma in a final cohort comprising 669 570 participants.

We also gathered 1996–2002 mortality data from the National Statistical Office. Subjects without cancer were followed until 31 December 2002; the follow-up period for each cancer case was defined as the interval between enrollment and diagnosis.

### Statistical analysis

The Cox proportional hazard regression model was used to estimate the relative risk of gastric cancer by subsite according to smoking status and alcohol intake (first adjusted only for age, then adjusted for other potential risk factors). Every model included the length of follow-up as a time-dependent covariate. The proportionality assumption was verified by inspecting hazard plots. The trends were assessed by assigning ordinal values for categorical variables. Non- and former smokers were excluded from the analysis of duration and intensity (cigarettes per day) when calculating the *P*-value for trend, as suggested by Lefforondré *et al* (2002). For alcohol consumption, trends were calculated among those who drank at least 1 g per day.

The interaction effects were evaluated by calculating an interaction term, that is, multiplying a dummy variable for smoking (current smoker=1, nonsmoker or former smoker=0) by one for alcohol consumption (drinks once per month=1, never drinks=0). Interactions between smoking and alcohol drinking were formally tested using the likelihood ratio method, comparing models with and without the interaction terms. We calculated a population-attributable risk (Rothman and Greenland, 1998) to assess the potential public health impact of smoking on gastric cancer by anatomic site, using the smoking prevalence data from the 1998 Korea Health Survey (Ministry of Health and Welfare, 1999). All confidence intervals (CIs) were at 95%, and a *P*-value of 5% was considered significant. All statistical analyses were performed with SAS version 9.1 (SAS institute, Cary, NC, USA).

## RESULTS

The 669 570 study cohort members were followed for an average 6.5 years, contributing a total of 4 353 317 person-years. The population was primarily middle aged (mean of 44 years) and had a low average BMI (23.6 kg m^−2^) with 29.4% of men over 25 kg m^−2^. At baseline, 53.5 and 71.8% of the men were current smokers and drinkers, respectively (Table 1). During follow-up, we identified 3452 new cases of gastric cancer, of which 127 (4%) were cardia and upper-third cancers, 2409 (70%) were distal gastric cancer and 1007 were unclassified. Some characteristics of the participants are presented in Table 1. Almost 90% of cases occurred in persons above 40 years.

Table 2 shows the adjusted relative risk (aRR) for gastric cancer in relationship to smoking and alcohol intake. The risk of cardia and upper-third gastric cancers was doubled or more among current smokers (aRR=2.2; 95% CI=1.4–3.5) when compared to those who had never smoked. For distal and total gastric cancer, the corresponding risks of current and never smokers were 1.4 (95% CI=1.3–1.6) and 1.5 (95% CI=1.4–1.6), respectively. Relative risks of gastric cancer increased with increasing numbers of cigarettes per day and years of smoking, although the trend was not statistically significant. The age-only adjusted risk estimates for smoking changed only slightly after adjusting for alcohol and other variables (see Materials and Methods), including alcohol intake (data not shown). We estimated the multivariate-adjusted population-attributable risks from cigarette smoking as 36.8% (95% CI 16.2–52.4) in cardia and upper-third gastric cancer, and 19.3% (95% CI 13.9–24.3) in distal gastric cancer; overall, 22.5% (95% CI 18.3–26.5) of gastric cancers were attributable to smoking.

Alcohol consumption was also associated with an increased risk after adjusting for smoking status (Table 3). The risks of distal and total gastric cancers were increased among patients who reported drinking at least 25 g of alcohol per day to 1.3 (95% CI 1.2–1.5) and 1.2 (95% CI 1.1–1.4), respectively, when compared to nondrinkers, and the *P*-value for trend was significant when only drinkers were considered. Although the risks of cardia and upper-third gastric cancer were increased among drinkers, these were not significant.

The independent and joint effects of smoking and alcohol intake on risks by gastric subsite are examined in Table 4. Smoking over 20 cigarettes per day combined with alcohol consumption exceeding 25 g per day was associated with a nearly five-fold increased risk of cardia and upper-third gastric cancer (HR=4.5, 95% CI =1.7–11.9), and a two-fold increased risk of distal gastric cancer compared to nonusers. The interaction between smoking and alcohol drinking was not statistically significant for total gastric cancer (*P*=0.48), cardia and upper-third cancer (*P*=0.68) or distal cancer (*P*=0.89).

## DISCUSSION

Smoking and alcohol use were associated with gastric cancer risk by anatomic subsite in this large cohort study. Current smokers showed elevated risks, higher in cardia and upper-third than in distal gastric cancer among current smokers. Furthermore, we found that the risk of cancer increased with the number of cigarettes smoked per day and years of smoking. Positive associations were also found with alcohol consumption, though for cardia and upper-third gastric cancers this was not significant. The results of the multinomial logistic analysis were similar to those of a Cox proportional hazard regression: the incidence of cardia and upper-third gastric cancer was 2.2 times higher for current smokers than for never smokers, and 1.4 times for distal gastric cancer (data not shown). Combined exposure to high levels of tobacco and alcohol further increased the risk estimates.

Although smoking is well recognised as a moderate risk factor, few population-based cohort studies have been conducted for gastric subsites (Sasazuki *et al*, 2002; Koizumi *et al*, 2004; Sjödahl *et al*, 2006). In the last 5 years, seven case–control studies have also been reported: five of them (Gammon *et al*, 1997; Zaridze *et al*, 2002) observed a higher risk for cardia cancers, while two did not (Brenner *et al*, 2002). We found moderately strong associations between smoking and gastric cancer in both cardia and upper third, and distal locations. Several dose–response associations were also suggested, adding to evidence of a causal association.

Although a significant association of cardia cancer with alcohol has been reported (Inoue *et al*, 1994), most studies have not confirmed this (Okabayashi *et al*, 2000; Sasazuki *et al*, 2002; Zaridze *et al*, 2002). We found that alcohol intake was significantly related to an increased risk both of gastric cancer as a whole and of the distal stomach. By contrast, the positive association for cardia and upper-third cancers was not significant, perhaps because of the relatively small numbers.

Several potential limitations of our study resulted from the use of data collected as part of an insurance plan. First, the self-reported smoking and alcohol details were not validated, and the amount smoked per day was classified only as ‘1–9’ and ‘20 or more’ on the 1996 questionnaire. Therefore, we could not examine, for example, 15–24 cigarettes smoked daily to cover the effect of rounding to a common value.

Second, our study cohort was not representative of all Koreans. Although enrollment in the NHI Program is largely mandatory for Koreans, our study covered only employed persons (government employees and teachers) and their families, and consequently, may have under-represented heavy users of alcohol and tobacco. However, follow-up should be essentially complete because of our using record linkage with unique personal identifiers to national databases.

Third, we lacked information on *Helicobacter pylori*, a strong risk factor for gastric cancer (International Agency for Research on Cancer, 1994). In Korea, the reported prevalence of *H*. *pylori* IgG antibody among males above 40 years is 77–83% (Shin *et al*, 2005a; Kim *et al*, 2006). Moreover, a nonsignificant increased risk for gastric cancer associated with the presence of *H*. *pylori* was observed among subjects in a longer than 5-year follow-up study in Korea (Shin *et al*, 2005a). In addition, evidence has shown that the association of smoking and gastric cancer is independent of *H*. *pylori* infection (Siman *et al*, 2001; Sasazuki *et al*, 2002). Given these findings, the lack of *H*. *pylori* data is unlikely to be an important issue for interpreting our findings.

Fourth, no detailed information on nutritional factors was available, including the intake of antioxidative vitamins, which might have a protective effect against gastric cancer (Kono and Hirohata, 1996).

In our study, cardia and upper-third gastric cancer was more strongly related to smoking status than distal gastric cancer, while alcohol consumption may be associated with an increased risk of distal and total gastric cancer. Larger numbers of cardia gastric cancer, however, would be needed to investigate a dose–response relationship reliably.

## Figures and Tables

**Table 1:** Distribution of cardia and upper-third, and distal gastric cancer by baseline demographic characteristics of the cohort

			**Gastric cancer**					
	**Cohort members**		**Cardia and upper third**		**Distal**		**Total^a^**	
	**(PY=4 353 317)**		**(PY=451)**		**(PY=8349)**		**(PY=12 242)**	
**Characteristic**	***n***	**%**	***n***	**%**	***n***	**%**	***n***	**%**
Total	669 570		127		2318		3452	
								
*Age at entry (years)* b	44.0	8.9	52.0	8.4	51.6	8.0	51.4	8.2
⩽39	249 932	37.0	12	9.4	234	10.1	357	10.3
40–49	229 403	34.3	33	26.0	575	24.8	891	25.8
50–59	159 958	23.9	59	46.5	1130	48.7	1632	47.3
⩾60	32 277	4.8	23	18.1	379	16.4	572	16.6
								
*Body mass index* (*kg* *m*^−*2*^)^b^	23.6	2.6	24.1	2.7	23.6	2.7	23.6	2.7
⩽22.9	274 971	41.1	42	33.1	853	36.8	1455	42.1
23.0–24.9	197 558	29.5	40	31.5	725	31.3	1030	29.8
⩾25.0	197 041	29.4	45	35.4	640	27.6	967	28.0
								
*Smoking status*								
Never	212 900	31.8	24	18.9	616	26.6	901	26.1
Former	98 229	14.7	24	18.9	405	17.5	593	17.2
Current	358 441	53.5	79	62.2	1297	56.0	1958	56.7
								
*Cigarettes per day* c								
⩽19	281 672	78.8	60	76.9	1027	79.7	1535	79.1
⩾20	75 567	21.2	18	23.1	261	20.3	406	20.9
								
*Smoking duration (years)* c								
⩽19	196 575	56.5	29	38.2	391	31.4	589	31.3
20–29	103 392	29.7	21	27.6	431	34.6	652	34.6
⩾30	47 673	13.7	26	34.2	424	34.0	642	34.1
								
*Alcohol intake* (*g* *day*^−*1*^)								
0	188 830	28.2	29	22.8	661	28.5	999	28.9
1–14.9	198 998	29.7	36	28.3	633	27.3	946	27.4
15.0–24.9	124 711	18.6	31	24.4	430	18.6	644	18.7
⩾25	157 031	23.5	31	24.4	594	25.6	863	25.0
								
*Preference for salty food*								
Low	274 971	41.1	12	9.4	335	14.5	511	14.8
Normal	197 558	29.5	82	64.6	1535	66.2	2261	65.5
Salty	197 041	29.4	33	26.0	448	19.3	680	19.7

PY=person-year.

a Including cardia and upper-third (C16.0–16.1), distal (C16.2–16.7), mixed site (C16.8) and site not otherwise specified (C16.9) gastric cancer.

b Data are means±standard deviation or number of cases and percentage.

c For current smokers.

**Table 2:** Multivariate relative risk by smoking habit for gastric cancer according to anatomic subsite

		**Gastric cancer**					
		**Cardia and upper third**		**Distal**		**Total^a^**	
	**No. of subjects**	***n***	**aRR^b^**	***n***	**aRR^b^**	***n***	**aRR^b^**
*Smoking status*							
Never	212 900	24	1.0	616	1.0	901	1.0
Former	98 229	24	1.9 (1.1–3.3)	405	1.3 (1.2–1.5)	593	1.3 (1.2–1.5)
Current	358 441	79	2.2 (1.4–3.5)	1297	1.4 (1.3–1.6)	1958	1.5 (1.4–1.6)
							
*Cigarettes per day*							
Never	212 900	24	1.0	616	1.0	901	1.0
1–19	281 672	60	2.3 (1.4–3.7)	1027	1.4 (1.3–1.6)	1535	1.5 (1.4–1.6)
⩾20	75 567	18	2.5 (1.3–4.7)	261	1.4 (1.2–1.6)	406	1.5 (1.3–1.7)
*p*_trend_ c			0.1953		0.3567		0.1223
							
*Smoking duration*							
Never	212 900	24	1.0	616	1.0	901	1.0
⩽19 years	196 575	29	2.9 (1.6–5.1)	391	1.4 (1.2–1.6)	589	1.4 (1.3–1.6)
20–29 years	103 392	21	1.9 (1.0–3.4)	431	1.4 (1.3–1.6)	652	1.5 (1.3–1.6)
⩾30 years	47 673	26	2.4 (1.3–4.2)	424	1.5 (1.3–1.7)	642	1.5 (1.4–1.7)
*p*_trend_ c			0.3387		0.8958		0.6302

aRR=adjusted relative risk.

a Including cardia and upper-third (C16.0–16.1), distal (C16.2–16.7), mixed site (C16.8) and site not otherwise specified (C16.9) gastric cancer.

b 95% confidence intervals from multivariate Cox proportional models after adjusting for age, body mass index, alcohol intake and preference for saltiness in food.

c Trend calculated among current smokers.

**Table 3:** Multivariate relative risk by alcohol consumption for gastric cancer according to anatomic subsite

		**Gastric cancer**					
		**Cardia and upper third**		**Distal**		**Total^a^**	
	**No. of subjects**	***n***	**aRR^b^**	***n***	**aRR^b^**	***n***	**aRR^b^**
*Alcohol consumption* (*g* *day*^−*1*^)							
0	188 830	29	1.0	661	1.0	999	1.0
1–14.9	198 998	36	1.3 (0.8–2.1)	633	1.0 (0.9–1.2)	946	1.0 (0.9–1.1)
15.0–24.9	124 711	31	1.7 (1.0–2.8)	430	1.2 (1.0–1.3)	644	1.1 (1.0–1.3)
⩾25	157 031	31	1.3 (0.8–2.2)	594	1.3 (1.2–1.5)	863	1.2 (1.1–1.4)
							
*p*_trend_ c			0.5914		0.0002		0.0001

aRR=adjusted relative risk.

a Including cardia and upper-third (C16.0–16.1), distal (C16.2–16.7), mixed site (C16.8) and site not otherwise specified (C16.9) gastric cancer.

b 95% confidence intervals from multivariate Cox proportional models after adjusting for age, body mass index, smoking and preference for saltiness in food.

c Trend calculated among those who drank at least 1 g day^−1^.

**Table 4:** Smoking, alcohol and risk of gastric cancer by subsite

**Exposure status**			**Gastric cancer**					
			**Cardia and upper third**		**Distal**		**Total^a^**	
**Smoking**	**Alcohol**	**No. of subjects**	***n***	**aRR^b^**	***n***	**aRR^b^**	***n***	**aRR^b^**
Never smoked	Never	88 431	8	1.0	270	1.0	397	1.0
Never smoked	<25 g day^−1^	96 063	15	2.0 (0.9–4.8)	255	1.0 (0.9–1.2)	378	1.0 (0.9–1.2)
Never smoked	⩾25	28 406	1	0.4 (0.1–3.4)	91	1.3 (1.0–1.6)	126	1.2 (1.0–1.5)
<20 cigarettes per day	Never	56 786	11	2.5 (1.0–6.2)	235	1.5 (1.3–1.8)	350	1.5 (1.3–1.8)
<20 cigarettes per day	<25 g day^−1^	149 534	31	3.1 (1.4–6.9)	499	1.5 (1.3–1.7)	758	1.5 (1.3–1.7)
<20 cigarettes per day	⩾25	75 352	18	3.7 (1.6–8.6)	293	1.8 (1.5–2.2)	427	1.8 (1.6–2.1)
⩾20 cigarettes per day	Never	16 585	2	1.5 (0.3–7.3)	44	1.0 (0.8–1.4)	85	1.4 (1.1–1.7)
⩾20 cigarettes per day	<25 g day^−1^	27 572	7	3.9 (1.4–10.8)	93	1.6 (1.6–2.0)	145	1.7 (1.4–2.0)
⩾20 cigarettes per day	⩾25	31 410	9	4.5 (1.7–11.9)	124	2.0 (1.6–2.5)	176	1.9 (1.6–2.3)

aRR=adjusted relative risk.

a Including cardia and upper-third (C16.0C16.1), distal (C16.2C16.7), mixed site (C16.8) and site not otherwise specified (C16.9) gastric cancer.

b Adjusted risk ratios and 95% confidence intervals from multivariate Cox proportional models after adjusting for age, body mass index and preference for saltiness in food.
